# Exploring the Cost of eLearning in Health Professions Education: Scoping Review

**DOI:** 10.2196/13681

**Published:** 2021-03-11

**Authors:** Edward Meinert, Jessie Eerens, Christina Banks, Stephen Maloney, George Rivers, Dragan Ilic, Kieran Walsh, Azeem Majeed, Josip Car

**Affiliations:** 1 Department of Primary Care and Public Health Imperial College London London United Kingdom; 2 Centre for Health Technology University of Plymouth Plymouth United Kingdom; 3 Department of Physiotherapy Monash University Melbourne Australia; 4 Faculty of Business and Economics Monash University Melbourne Australia; 5 Medical Education Research and Quality School of Public Health and Preventive Medicine Monash University Melbourne Australia; 6 BMJ Knowledge Centre BMJ Learning London United Kingdom

**Keywords:** education, distance education, professional education, online education, online learning, costs and cost analysis, economics

## Abstract

**Background:**

Existing research on the costs associated with the design and deployment of eLearning in health professions education is limited. The relative costs of these learning platforms to those of face-to-face learning are also not well understood. The lack of predefined costing models used for eLearning cost data capture has made it difficult to complete cost evaluation.

**Objective:**

The key aim of this scoping review was to explore the state of evidence concerning cost capture within eLearning in health professions education. The review explores the available data to define cost calculations related to eLearning.

**Methods:**

The scoping review was performed using a search strategy with Medical Subject Heading terms and related keywords centered on eLearning and cost calculation with a population scope of health professionals in all countries. The search was limited to articles published in English. No restriction was placed on literature publication date.

**Results:**

In total, 7344 articles were returned from the original search of the literature. Of these, 232 were relevant to associated keywords or abstract references following screening. Full-text review resulted in 168 studies being excluded. Of these, 61 studies were excluded because they were unrelated to eLearning and focused on general education. In addition, 103 studies were excluded because of lack of detailed information regarding costs; these studies referred to cost in ways either indicating cost favorability or unfavorability, but without data to support findings. Finally, 4 studies were excluded because of limited cost data that were insufficient for analysis. In total, 42 studies provided data and analysis of the impact of cost and value in health professions education. The most common data source was total cost of training (n=29). Other sources included cost per learner, referring to the cost for individual students (n=13). The population most frequently cited was medical students (n=15), although 12 articles focused on multiple populations. A further 22 studies provide details of costing approaches for the production and delivery of eLearning. These studies offer insight into the ways eLearning has been budgeted and project-managed through implementation.

**Conclusions:**

Although cost is a recognized factor in studies detailing eLearning design and implementation, the way cost is captured is inconsistent. Despite a perception that eLearning is more cost-effective than face-to-face instruction, there is not yet sufficient evidence to assert this conclusively. A rigorous, repeatable data capture method is needed, in addition to a means to leverage existing economic evaluation methods that can then test eLearning cost-effectiveness and how to implement eLearning with cost benefits and advantages over traditional instruction.

## Introduction

Significant investment is necessary to adapt and expand global health care staff to transition to the medical challenges of the 21st century. The demands on the workforce range from an aging population and emphasis on chronic disease management [1] to access to primary care, where there is a direct link to the cost of training medical personnel. Primary care depends more heavily on public sector investment than other medical specialties, and scarce resources limit the number of personnel who can be trained [2]. As one example, with the increasing cost of delivery of care within the United Kingdom, the National Health Service has recognized that medical providers must take a greater role in education and training [3]. Creating production efficiencies in education and training may assist with the supply of medical personnel to support clinical skills and applied health-related skills. eLearning, defined as “an approach to teaching and learning, representing all or part of the educational model applied, that is based on the use of electronic media and devices as tools for improving access to training, communication and interaction and that facilitates the adoption of new ways of understanding and developing learning” [4], presents a possible opportunity to change and optimize training by providing a scalable means for instruction, thus reducing the costs necessary in delivery and implementation. 

A potential critical opportunity of eLearning is the long-term efficiency gain in its delivery model in contrast to other forms of instruction; however, the costs to develop eLearning are significant when executed to a high standard [5]. To achieve better cost management of eLearning and ensure scale-up and adoption, data are required to identify the factors that influence eLearning design and production. Research on the use of eLearning in medicine suggests that measurement of costs in studies is often inconsistent [6]. Therefore, the aim of this scoping review was to provide a broad overview of the state of evidence concerning measurement of costs in eLearning. Understanding these costs will enable better planning in the design and production of eLearning.

## Methods

### Design

Scoping reviews are a form of rapid knowledge synthesis that identify the sources and evidence available to address research questions in a systematic manner. The established scoping review methodology by Levac et al [7] was chosen for this review, as the research question aims to provide a broad understanding of the literature available in this field to ultimately inform subsequent reviews or research agendas.

### Identifying the Relevant Research Question

To establish a comprehensive understanding of the costs [8] associated with eLearning, we conducted a scoping review [7,9] to assess the available literature that quantifies the cost to deliver eLearning in health professions education. For the purpose of this review, cost is defined as the total costs (direct and indirect) from inception to deployment, including the design, development, and delivery (or implementation). Within the study analysis, we attempt to analyze how these costs have been reported by studies, with an understanding that separate factors and sources of these total costs may or may not be reported. Factors influencing these costs could, for example, include the level of experience of the teams producing content. This aggregate grouping of studies will impact the way studies are compared to each other and should be taken into account when reading this review, as other study themes or classifications could impact interpretation of results. The research question under investigation is: What is known in the literature about cost calculations related to eLearning in health professions education in regard to (a) practical cost analysis, with respect to cost per learner and comparison to face-to-face instruction; and (b) the choices in practice of costing methods and models? A secondary question is: How has the publication frequency of this field developed over time?

These questions were derived using the PICO (Population, Intervention, Comparison, Outcome) framework [10]. In this review, the *population* is defined as learners in health professions in all countries; this decision was made to ensure comprehensive coverage of all health professionals to best understand the state of evidence internationally. The *intervention* instrument being evaluated is eLearning in health professions education (inclusive of various forms of training, including basic and advanced continuing professional development, university-level training, patient education, and various other training forms provided by an equally broad group of education training providers). The *comparison* used in this study is the evaluation of costs between eLearning, other methods of instruction such as face to face, and alternate approaches to eLearning, or studies that do not make use of a comparator. The *outcome* was quantification and analysis of the difference in costs between and within the implementations. We defined costs from cost calculations used in economic evaluation, including cost-consequence analysis, cost-minimization analysis, cost-effective analysis, cost-utility analysis, and cost-benefit analysis [11].

### Identifying Relevant Studies

Following consultation with an information scientist at the Imperial College London Medical School Library on literature search approaches, a search of the following databases was performed in December 2015 and repeated in December 2018: PubMed, Scopus, Education Resource Information Centre (ERIC), Web of Science, Embase, Global Health, Health Management Information Consortium (HMIC), Prospero, and OVID. In a second search, which was completed in December 2018, new papers were added to the original dataset but did not undergo exhaustive data charting; the data included provided a high-level summary of contents and relevance to previously categorized themes (these papers can be identified as studies from 2016 to 2018).

The search strategy included use of Medical Subject Heading terms and related keywords centered on eLearning and cost calculation with a population scope of health professionals in all countries. The search was limited to English-language studies. There was no restriction placed on literature publication date; although online technologies have changed rapidly over a short period of time, the authors felt that to provide a comprehensive overview of the literature, it would be useful to first explore research with no date restriction. The primary research questions were kept broad to ensure that there would be inclusion of all studies that recorded the costs to deliver eLearning globally. A high-level summary of the search strategy is detailed in Textbox 1; a full summary of the search strategy used per database is detailed in Multimedia Appendix 1.

**Sample search terms.**

Cost-related terms

- Costs and Cost Analysis [Medical Subject Heading (MeSH) terms]
- Cost-benefit analysis [MeSH Terms]
- Costs and cost analysis [MeSH Terms]
- Cost*
- Economic*

Learning-related terms

- Learning [MeSH Terms]
- eLearning
- Blended learning
- Online learning

### Study Selection

Following the process used in this scoping review method, study selection was based on study identification with data centered on studies that identified cost factors and variables in health professions education eLearning. The literature was reviewed independently by two researchers (JE and EM) to identify articles. A third researcher (CB) adjudicated disagreements when necessary. Article abstracts were first scanned for relevance to the research question and then full articles were downloaded to verify appropriateness. The inclusion criteria included studies and reviews that examined eLearning in health professions education, and captured data concerning design, development, and production costs. Papers that provided synthesis or editorializing of issues without data (ie, opinion pieces and commentaries) were excluded (Multimedia Appendix 2).

### Charting the Data

The definition of cost in this review is centered on the hypothesized cost savings derived from a possible reduction in labor costs through scaling teaching via digital technology; cost was defined as the production and delivery costs (direct and indirect) of online learning [12]. Studies included were classified to explore different ways of comparing and analyzing factors influencing these costs. Studies were chartered into two groups: (1) studies detailing costs for eLearning implementations and (2) studies with detailed costing methods (approaches to capture costs) for eLearning but without implementation of specific data. Group 1 was further charted into two separate groups: (1) studies with comparison to other learning types and (2) studies without a comparator. For these two subcategories, we excluded studies disclosing that the cost data provided were incomplete.

### Collating, Summarizing, and Reporting the Results

Each study was reviewed individually to understand the implementation aspects of each reported eLearning instance. The studies were then summarized into four categories: (1) studies that detail eLearning costs without a comparator, (2) studies that detail eLearning costs with a comparator, (3) related data from two related systematic reviews, and (4) studies that detail costing approaches. The results are presented as a narrative summary of the principal aspects of each study organized via main classification themes to present evidence that can inform the development and deployment of eLearning by defining the factors that influence implementation costs and the criteria that should be used to explore cost optimization.

## Results

### Overview of Included Studies

**Figure 1:**
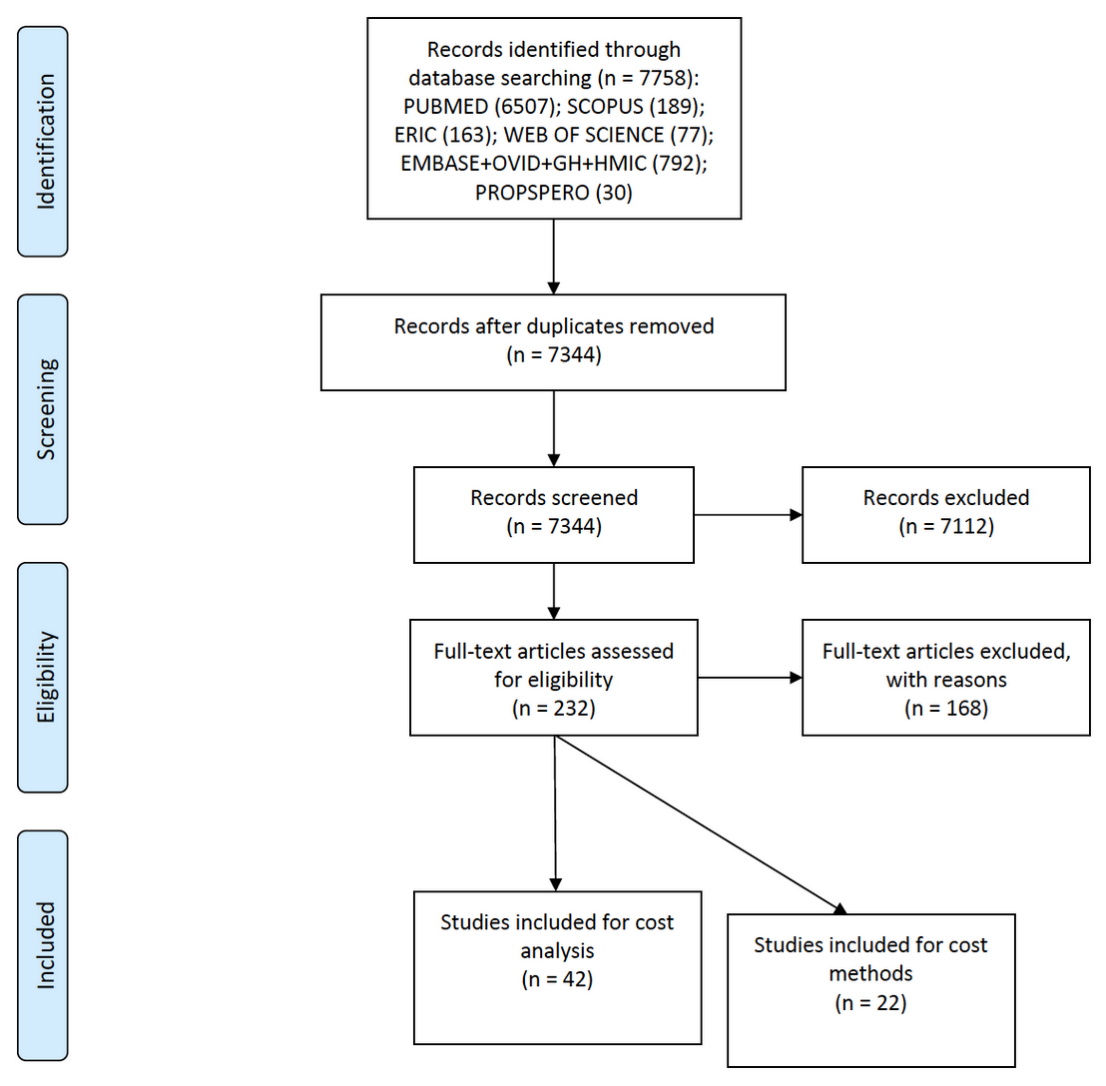
PRISMA (Preferred Reporting Items in Systematic Reviews and Meta-Analyses) flow diagram of search and screening for costs of eLearning implementation.

In total, 7344 articles were returned from the search of the literature (Figure 1). Of these, 232 were relevant to associated keywords or abstract references to cost following screening. Full-text review resulted in 168 studies being excluded. Of these, 61 studies were excluded because they were unrelated to eLearning and focused on general education. In addition, 103 studies were excluded because of lack of detailed information regarding costs; these studies referred to cost in ways either indicating cost favorability or unfavorability, but without data to support findings. Finally, 4 studies were excluded because of limited cost data insufficient for analysis. In total, 42 studies (Table 1) provided data and analysis of the impact of cost and value in health professions education. Completeness of data extracted varied, which resulted in some datasets in the final inclusion data charts to be designated as not available/applicable to reflect inability to abstract usable information; however, these studies remained within the inclusion set because of partial data that contributed to the narrative analysis. These studies contrasted to studies excluded at the earlier screening stage because of cost being a secondary outcome of the investigation and the cost data being of greater focus than those of the excluded studies. The most common data source was the total cost of training (n=29). Other sources included cost per learner, meaning the cost per student (n=13). The population most frequently cited was medical students (n=15), although a group of articles focused on multiple populations (n=12). A further 22 studies provide details of costing approaches for the production and delivery of eLearning. These studies offer insight into the ways that eLearning has been budgeted and project-managed through implementation.

**Table 1:** Studies that provide costs for eLearning implementation.^a^

Reference	Year	Comparison	Study design	Subject	Cost source	HCP^b^ population
Allan et al [13]	2008	None	Case	Evidence-based medicine	Total cost	Clinicians
Bandla et al [14]	2012	None	Case-control	Sleep medicine	Total cost	Medical students
Berger et al [15]	2009	Face to face	Case- control	Patient education	Per learner	Nurses
Butler et al [16]	2013	None	RCT^c^	Behavior change counseling	Per learner	Clinicians, nurses
Choi et al [17]	2008	Other learning	Case	Surgical anatomy	Total cost	Medical students
Collins et al [18]	2018	None	Course review	Nutrition	Total cost	AHPs^d^, medical students
Downer et al [19]	2018	None	Case	Leadership and management in health	Total cost	AHPs, medical students, clinicians
Dumestre et al [20]	2014	Other learning	Systematic review	Microsurgical skill acquisition	Per learner	Clinicians, medical students
Glasbey et al [21]	2017	Face to face	Case	Surgical training	Total cost	Medical students
Grayson et al [22]	2018	None	Longitudinal	Hand hygiene	Total cost	AHPs, medical students, clinicians
Hardwick et al [23]	2011	None	Case	Pathology	Total cost	Clinicians
Jerin and Rea [24]	2005	None	Case	Emergency medicine	Per learner	AHPs
Joshi and Perin [25]	2012	Other learning	Case	Public health informatics	Total cost	AHPs
Kaufman [26]	2010	None	Case	Treatment of diabetes	Per learner	Patients (patient education used by HCP)
Knapp et al [27]	2011	Face to face	Case	HIV detection	Total cost	AHPs, clinicians
Kumpu et al [28]	2016	Face to face	Case	Global health	Total cost	AHPs, medical students, clinicians
Letterie et al [29]	2003	None	Literature review	Computer-assisted medical education	Total cost	AHPs, medical students, clinicians
Likic et al [30]	2013	None	Cohort	Rational therapeutics	Total cost	Medical students
Manring et al [31]	2011	None	Case	Psychotherapy	Total cost	Clinicians
McConnell et al [32]	2009	None	Case	Pharmacy CPD^e^	Per learner	Pharmacists
McDuffie et al [33]	2011	None	Case	Experiential pharmacy training	Per learner	Pharmacists
Moreno-Ger et al [34]	2010	No Intervention	Case	Practical skills simulation	Per learner	Medical students
Nickel et al [35]	2015	Other learning	RCT	Laparoscopic cholecystectomy	Total cost	Medical students
Nicklen et al [36]	2016	None	Case	Physiotherapy	Total cost	Undergraduate AHPs
Padwal et al [37]	2017	Other learning	RCT	Weight management	Total cost	Patients (patient education used by HCP)
Padwal et al [38]	2013	Other learning	RCT	Weight management (study protocol)	Total cost	Patients (patient education used by HCP)
Palmer et al [39]	2015	None	Case	Clinical skills	Total cost	Medical students
Pentiak et al [40]	2013	None	Clinical review	Surgical skills	Per learner	Clinicians
Perkins et al [41]	2012	Face to face	RCT	Advanced life support training	Per learner	AHPs
Reeves et al [42]	2013	Other learning	Literature review	Interprofessional education	Total cost	AHPs
Schopf and Flytkjær [43]	2011	None	Case	Interprofessional training -dermatology	Total cost	Clinicians, nurses
Shepler [44]	2014	None	Cohort	Advanced pharmacy practice experience	Total cost	Pharmacy students
Sivamalai et al [45]	2011	None	Case	Pathology	Total cost	Medical students
Spanou et al [46]	2010	Face to face	RCT (protocol)	Behavior change counseling	Total cost	Clinicians, nurses
Stansfeld et al [47]	2015	Other learning	RCT	Employee well-being	Total cost	AHPs
Stromberg et al [48]	2012	None	Cohort	Heart failure nursing	Total cost	Nurses
Thomas et al [49]	2010	None	Case	Family planning	Total cost	AHPs
de Ruijter et al [50]	2015	None	Case	Business engineering; surgical technician	Total cost	Medical students
Weiss et al [51]	2011	Other learning	Cohort	Antibiotic prescribing	Total cost	Clinicians, pharmacists
Williams et al [52]	2009	None	Cohort	Practice-based research networks	Per learner	Clinicians
Young et al [53]	2017	None	Case	Research skills	Per learner	AHPs
Zhou et al [54]	2018	None	Case	Resource stewardship	Per learner	Medical students, clinicians

^a^These studies were all assigned the prefix “INC,” indicating that this group was inclusive of both comparator and noncomparator studies (for eLearning costs); the combination of the prefix and study number can be used to provide a unique ID to refer to studies.

^b^HCP: health care provider.

^c^RCT: randomized controlled trial.

^d^AHPs: allied health professionals.

^e^CPD: continuing professional development.

### Studies Describing eLearning Costs Without a Comparator

Twenty-two studies [13,16,19,22,23,26,30-34,39,40,43-45,48, 50,52-55] provided analysis of implementation costs in eLearning without comparison to other learning platforms. These studies primarily reported total costs and cost per learner (Table 2). The studies suggested that eLearning should be less costly than face-to-face learning; however, without a comparator, it is not possible to substantiate these claims. Despite these deficiencies, these studies provide varying means of cost calculation across different forms of instructional design.

**Table 2:** Studies that detail eLearning costs without a comparator.^a^

Reference	Year	Instructional design	Sample size (N)	Total cost (US $)	Cost per learner (US $)	Notes
Allan et al [13]	2008	Asynchronous, blended	304	8209	24	No blended learning cost
Butler et al [16]	2013	Blended	80	2075	26	No explicit cost methodology/technique described
Downer et al [19]	2018	Asynchronous	53	23,000	394	No explicit cost methodology/technique described
Grayson et al [22]	2018	Asynchronous	1,989,713	N/A^b^	0.04	Provided aggregate cost per leaner
Kaufman [26]	2010	Asynchronous	787	N/A	1453	Reported overall cost per learner
Hardwick et al [23]	2011	Asynchronous	N/A	N/A	N/A	Provided cost modeling approach
Likic et al [29]	2013	Asynchronous	393	10,000	23	Use of online course deemed lower cost than face-to-face problem-based learning
Manring et al [31]	2011	Blended	35	5250	137	Only costs of physical implementation
McConnell et al [32]	2009	Asynchronous	8120	610	0.07	No explicit cost methodology/technique described
McDuffie et al [33]	2011	Blended	382	N/A	21	No explicit cost methodology/technique described
Moreno-Ger et al [34]	2010	Asynchronous	400	2630	6	No explicit cost methodology/technique described
Palmer et al [39]	2015	Synchronous	9	5000	506	No explicit cost methodology/technique described
Pentiak et al [40]	2013	Asynchronous	N/A	32,685	N/A	Total curriculum delivery
Schopf and Flytkjær [43]	2011	Asynchronous	88	84,229	858	No explicit cost methodology/technique described
Shepler [44]	2014	Asynchronous	580	N/A	N/A	US $148 savings per intervention
Sivamalai et al [45]	2011	Asynchronous	200	392,468	1782	Cost of digital microscopy 1/3 cost of physical microscopy
Stromberg et al [48]	2012	Asynchronous	183	N/A	N/A	Total cost reduction compared over previous methods
Thomas et al [49]	2010	Asynchronous	273	21,000	70	No explicit cost methodology/technique described
de Ruijter et al [50]	2015	Asynchronous	803	44,986	49	No explicit cost methodology/technique described
Williams et al [52]	2009	Asynchronous	103	3732	33	No explicit cost methodology/technique described
Young et al [53]	2017	Asynchronous	679	N/A	38	Did not report total cost
Zhou et al [54]	2018	Asynchronous	48	N/A	148	Did not report total cost

^a^These studies are given the prefix “SUM” to indicate that this group represents a summary of costs without a comparator; the prefix and number can be used to provide a unique ID to refer to studies.

^b^N/A: not available/applicable.

The studies in this set engaged the scope of the review question focused on the costs associated with eLearning in health professions education but lacked the comparison variable of the PICO framework. Although these studies suggest that implementation of eLearning could provide self-reported high value through low-cost delivery, and thus cost-effectiveness, they offer no comparative framework to justify these assertions. Among the studies that quantify eLearning costs, three groups emerged. The first included studies demonstrating that eLearning was of low cost but had no or limited evidence of self-reported educational impact [13,16]. The second group demonstrated that eLearning was of low cost and had a high self-reported education impact [23,30-34,43-45,48-50,52-54]. A third group [19,22,26,39,40] demonstrated that eLearning was of high cost and had a high self-reported educational impact.

Allan et al [13] and Butler et al [16] present examples of low-cost eLearning delivery but without demonstrated educational impact, with low cost in these studies presented from the perspective of the cost per learner. In Allan et al [13], the key research question was whether this research group could implement an evidence-based medicine curriculum for clinicians. Although quantifying costs was an aspect of the reported results, like many of the studies included in this review, it was not a primary focus and was done so in an informal fashion without explicit unit cost breakdown or listing of all of the components that would impact learning production. In contrast to the use of a comprehensive program including multiple forms of learning and the establishment of a learning community, Butler et al [16] made use exclusively of blended learning in a course. They revealed that the complete training costs are not captured when creating online or blended courses in primary care. Despite comprehensively capturing unit costs of delivery in the implementation of the study (by providing segmentation of costs across administrators, actors, trainers, clinicians, nurses, and costs per practice), their study treated eLearning as a single-group cost reflecting the time per participant to complete the eLearning; however, there was no accounting of the required system implementation time and production time for the creation of eLearning. Similar to Allan et al [13], Butler et al [16] highlight cost omissions that are endemic in studies included in this review.

A second group of studies demonstrate eLearning as having low cost and high educational impact [23,30-34,43-45,48-50,52-54]. Of this set, Likic et al [30], McConnell et al [32], McDuffie et al [33], de Ruijter et al [50], Moreno-Ger et al [34], Thomas et al [49], Williams et al [52], and Young et al [53] each represent online courses making use of asynchronous online learning at low cost per learner (below US $68/learner). The key issue among the studies in this literature cluster is that although they may provide evidence of low cost per learner, without a comparison point to comparable face-to-face delivery, there is no way to assert with any certainty that eLearning is a lower-cost option.

The final group of studies in this set [19,22,26,39,40] indicated that eLearning was of higher cost and had high educational impact. This group shared similar data-recording issues as those from the previous set but also provide evidence to indicate the high start-up costs associated with eLearning production.

It is challenging to draw strong inferences based on an aggregation of the studies that summarize eLearning costs because of the different methods that were used in cost calculation, the difference in subjects instructed, the rapid changes in web platforms for learning, and other factors impacting the way costs were calculated. However, it is possible to observe some trends from this grouping. For pure online courses, the studies suggest that total costs per learner are low; however, there is often acknowledgment in the studies that not all implementation costs have been captured in the cost calculations. This lack of included costs, including sunk costs, indicates that reported costs are not accurate. Although some studies identified the costs that were not captured, many did not, and these gaps are only evident to researchers who have a background and understanding of the issues involved in the delivery of eLearning. Additionally, most studies are cases of specific instances of eLearning implementation, making it difficult to gauge what the results mean in contrast to face-to-face learning, and case study methods make it hard to generalize the results. Some studies indicated high total costs, but in those instances [40], the eLearning costs were embedded in total curriculum delivery.

### Studies Describing eLearning Costs With a Comparator

Seventeen studies [14,15,17,21,24,25,27,28,34-37,41,46,47,51] compared eLearning costs to those of face-to-face learning or other types of learning (Table 3). These comparative studies offered more evidence that the use of eLearning demonstrated cost efficiencies than did the studies in the previous group, which provided no comparative data.

**Table 3:** Studies that detail eLearning costs with a comparator.^a^

Reference	Year	Instructional design	Comparison	Sample size (N)	Cost of eLearning (US $)	Cost of face-to-face learning (US $)	Notes from study
Bandla et al [14]	2012	Asynchronous online	Face to face	173	21,752	21,752	N/A^b^
Berger et al [15]	2009	Blended	Face to face	1661	4	110	Cost per learner
Choi et al [17]	2008	Asynchronous online	Other learning	34	N/A	N/A	Provided costs of online platforms without complete cost comparison
Glasbey et al [21]	2017	N/A	N/A	570	N/A	N/A	Online curriculum embedded; core costs not separated in study
Jerin and Rea [24]	2005	Asynchronous online	Asynchronous online	9353	3	52	Cost per learner
Joshi and Perin [25]	2012	Asynchronous online	Other learning	15	14,085	20,714	Online vs face-to-face total costs
Knapp et al [27]	2011	Asynchronous online	Face to face	91	157	4386	N/A
Kumpu et al [28]	2016	Blended	Face to face	28	2431	1054	N/A
Moreno-Ger et al [34]	2010	Asynchronous online	Face to face	400	7	2630	N/A
Nickel et al [35]	2015	Virtual reality	Other learning	84	3900	82,500	Virtual reality vs blended learning
Nicklen et al [36]	2016	Blended	Face to face	78	5904	6856	N/A
Padwal et al [37]	2017	Asynchronous online	Face to face	651	11,727	477,000	N/A
Padwal et al [38]	2013	Asynchronous online	Face to face	N/A	N/A	N/A	Protocol
Perkins et al [41]	2012	Blended	Face to face	3732	438	935	N/A
Spanou et al [46]	2010	Asynchronous online	Face to face	N/A	N/A	N/A	Protocol
Stansfeld et al [47]	2015	Asynchronous online	Face to face	350	N/A	N/A	Captured approach to total costs but incomplete comparison data to nononline approach
Weiss et al [51]	2011	Asynchronous online	Other learning	N/A	N/A	N/A	Cost reduction per inhabitant following education program

^a^These studies were given the prefix “COMP” to indicate that this group was a summary of costs with a comparator; the prefix and number can be used to provide a unique ID to refer to studies.

^b^N/A: not available/applicable.

The studies in this set can be divided into two groups: studies that demonstrated that eLearning was of lower cost but had no or limited evidence of self-reported educational impact, and studies that demonstrated that eLearning was of lower cost and had self-reported high educational impact [25,51].

Of the studies that demonstrated that eLearning was of lower cost and had a low education impact, the key data issue was that although these studies suggested that eLearning was lower cost, they consistently omitted key components in the design and production of eLearning, thereby creating an incomplete cost profile of the total costs of delivery. Two studies in this set demonstrated that eLearning was of lower cost and had a high education impact; although each study completed a full comparison demonstrating a reduction in costs (in some instances a dramatic reduction), the studies suffer from a lack of methodological consistency in the way they captured costs and evaluated effectiveness. As was the case in the previous set of study classifications, the continued differences in cost accounting, learning delivery platforms, and various forms of assessments make synthesis challenging.

### Literature Reviews That Quantify eLearning Costs

Two review studies [20,42] analyzed the use of training where eLearning was used as a delivery platform. Both studies revealed that there was a lack of sufficient evidence to analyze whether training methods using aspects of online learning were more pedagogically effective. The studies were also unable to provide findings that created a holistic understanding of associated cost ingredients. Dumestre et al [20] suggested that within the field of microsurgical training, there are many available methods of implementing instruction and that cost is the determining factor in what method is used by institutions. Reeves [42] performed a Cochrane systematic review protocol that included 15 studies. The review showed that due to the small number of studies (N=15) and the heterogeneity of interventions and outcome measures, it is not possible to draw inferences about the key elements of interprofessional education and its effectiveness. To make such evaluation possible, there must be implementation of cost-benefit analysis, and separation of review within specific professions and studies using qualitative methods to evaluate effectiveness. Although both studies were concerned with evaluation of the effectiveness of specific education training, the way they engaged with the literature review question was limited, as both studies collected limited information on eLearning and only gave broad summary generalizations about cost reductions in their respective field of focus. Costs were identified by looking at the total costs of the delivery of programs; however, because the costs were not described as units, it is not possible to examine the extent and quality of the results. There was no accommodation for differential timing or impact of the consequences of cost decisions. These issues are similar to the weakness in cost analysis of the other studies included in this review.

### Studies Describing Costing Approaches

Twenty-two studies [56-77] referenced economic evaluation (analyzing cost benefits or cost effectiveness) or used the ingredients method [78] to calculate costs in the production of eLearning (Table 4). Reflecting on the broader set of studies in this review, it is important to note that while many studies suggest the cost-effectiveness of eLearning, following completion of this review, we have only identified 5 cost-effectiveness analysis studies completed on eLearning. Regarding specific cost approaches, use of the ingredients method is referenced often in this set (12 times); however, the mechanisms for cost capture and subsequent project delivery management of production of learning within this group are inconsistent despite using the same methods.

**Table 4:** Studies detailing costing approaches or economic evaluation.

Reference	Year	Costing approach
Brown [56]	2014	Cost-benefit analysis
Buntrock et al [57]	2014	Cost-effectiveness analysis
Pettit et al [58]	2017	Ingredients cost method
Carlson et al [59]	2008	Ingredients cost method
Carpenter [60]	2016	Ingredients cost method
Chambers et al [61]	2017	Cost utility analysis
Chhabra et al [62]	2013	Cost-effectiveness analysis
Cousineau et al [63]	2008	Cost-effectiveness analysis
Curran et al [64]	2006	Ingredients cost method
Cook [65]	2014	Ingredients cost method
Delgaty [66]	2013	Ingredients cost method
Djukic et al [67]	2015	Ingredients cost method
Gallimore et al [68]	2012	Ingredients cost method
Isaacson et al [69]	2014	Ingredients cost method
Lonsdale et al [70]	2016	Cost-effectiveness analysis
Papadatou-Pastou et al [71]	2017	Multiple; survey of methods
Pardue [72]	2001	Ingredients cost method
Pickering and Joynes [73]	2016	Multiple; survey of methods
Rondags et al [74]	2015	Cost-effectiveness analysis
Sharma et al [75]	2018	Ingredients cost method
Tung and Chang [76]	2008	Perceived financial cost
Zary et al [77]	2006	Ingredients cost method

## Discussion

### Principal Findings

Our review was focused on identifying literature that would define the associated costs in the delivery of eLearning in health professions education. Broadly speaking, we were able to answer this question as we collected data that documented a trend of reported eLearning costs per learner and their general low cost. However, we have questions about how conclusive these data are because of the issue of consistency regarding cost data capture, the lack of standard mechanisms for cost data collection for online learning, and the lack of primary studies that focused on cost analysis as a primary research objective. Our review findings are consistent with views put forth in previous research that understanding of the relationship of cost in eLearning is not well developed [6,79,80]. The studies included provide a cross-section of various instances of eLearning across many disciplines in health professions education. This collection of studies allowed gaining a deeper understanding of the various ways in which eLearning is being used and the cost considerations when applying different platforms of education delivery. The key limitation of the included studies was the lack of consistency of methodology for cost analysis. Cost evidence provided by the included studies was challenging for the purposes of comparison due to these deficiencies.

### Strengths and Limitations

The strengths of this review are that it completed a comprehensive search of the major literature databases. The search question and the associated terms provided a sufficiently broad scope to ensure that there was coverage to any study that recorded cost and maintained relevance to the inclusion criteria. The search approach was designed in consultation with leading researchers who investigate cost in education, and the final results provide a rich background of materials to explore the issues associated with the research question.

There are four limitations to the process used in this literature review. First, as only English-language papers were searched, relevant foreign-language papers could have been excluded, in addition to the publication bias of health science papers for positive results. Additionally, industry literature was not explicitly searched in the search strategy, further adding to the limitation of study papers under review. Second, due to the inconsistency in capturing costs and lack of standardization in cost reporting, a meta-analysis for quantifying costs is not possible because of the lack of predefined costing models for eLearning used in standard ways across studies, the significant variance in the way costs are recorded, variant experimental methods with different outcome conclusions, and the variance in implementation between different eLearning types. Third, a significant limitation is that in comparing costs of eLearning within the included studies of the review, each study was treated equally, whereas the costs for a team new to eLearning production will likely be higher than those of an experienced team who have produced many courses. Additionally, reported costs could have been on segments of the production process, resulting in inconsistency in reporting. Further research could explore specific aspects of design, development, and delivery to allow for more refined comparison and analysis, including quantitative cost analysis such as that of fixed versus variable costs. In addition to this cost analysis, further work could explore the relationship between learning impact and associated effort as attributed to cost. Lastly, a significant limitation is that this review was rerun in December 2018 to update results from spring 2016 in an original scoping of the literature completed in December 2015, but detailed analysis of new studies identified from 2016 to 2018 are not included in the narrative of this review. Although the newly included studies are incorporated into the data tables, because of time constraints, further analysis of these new studies will be completed in a separate update of this review.

Therefore, the review could be strengthened by taking further measures to either refine the research question into a narrower scope or attempting cost modeling with accepted deficiencies. Nevertheless, the review as completed provides a comprehensive scope of the current evidence, and highlights a gap in the literature indicating a need for a protocol that can capture costs in eLearning interventions to allow a basis for comparison in similar educational subjects or across variant curriculum implementations. Such a protocol would provide a systematic mechanism for calculating online learning costs to allow for a basis of various forms of economic evaluation. This would assist course designers in understanding the total costs in delivery of eLearning and address the standardization issues incumbent with a lack of a standard as evidenced by this review.

### Conclusions

Although cost is a recognized factor in studies exploring eLearning design and implementation, the way cost is captured is inconsistent and is assessed in relation to a wide variety of factors or with an alternate study–related focus. Despite a perception that eLearning is more cost-effective than face-to-face instruction, there is not yet sufficient evidence to assert this conclusively. Among the many factors for considering implementing eLearning is the potential long-term cost-effectiveness of its delivery model in comparison to other education delivery formats. A rigorous, repeatable data capture method is needed, in addition to a means to leverage existing economic evaluation methods that can then test whether eLearning is cost-effective, and how to implement eLearning with cost benefits and advantages over traditional instruction. On the one hand, if proven to be more cost-effective, this could assist in addressing the high cost of delivering health professions education. On the other the hand, should evidence point the other way, having discrete data points will allow those involved in health education to identify ways to optimize costs in eLearning delivery to create cost efficiency. To evaluate and optimize cost in education delivery, there must be a rigorous standard through which to score and assess cost-effectiveness, which would enable analysis of whether investments are justified.

To gain a comprehensive understanding of the way cost impacts the deployment of eLearning in comparison to face-to-face instruction, a body of evidence that makes use of economic evaluation must be developed to allow for systematic analysis of how these results demonstrate the strengths and weaknesses of comparative cost delivery. This review has identified the limited use of economic evaluations to achieve this aim thus far. Moreover, even among studies that make use of cost summaries in their results, there is a lack of sufficient rigor to provide insight into the way in which these costs impact education delivery or to allow for comparisons to other forms of learning.

## Appendix

Multimedia Appendix 1 Full search strategy.

Multimedia Appendix 2 Eligibility stage search exclusions.

## Abbreviations

PICO: Population, Intervention, Comparison, Outcome
