# Supplementary material for: Exploring the Cost of eLearning in Health Professions Education: Scoping Review
Source: JMIR Med Educ. 2021 Mar 11;7(1):e13681. doi: 10.2196/13681 (PMC8081275; doi:10.2196/13681)
Supplement: Multimedia Appendix 1 [file mededu_v7i1e13681_app1.docx]

# Multimedia Appendix 1: Full Search Strategy

**Electronic database searches for peer-reviewed literature**

1. **PubMed**

(((‘Costs and Cost Analysis’[Mesh] OR (‘cost-benefit analysis’[MeSH Terms] OR (‘cost-benefit’[All Fields] AND ‘analysis’[All Fields]) OR ‘cost-benefit analysis’[All Fields] OR (‘cost’[All Fields] AND ‘effectiveness’[All Fields]) OR ‘cost effectiveness’[All Fields])) OR (‘cost-benefit analysis’[MeSH Terms] OR (‘cost-benefit’[All Fields] AND ‘analysis’[All Fields]) OR ‘cost-benefit analysis’[All Fields] OR (‘economic’[All Fields] AND ‘evaluation’[All Fields]) OR ‘economic evaluation’[All Fields])) OR ((‘cost-benefit analysis’[MeSH Terms] OR (‘cost-benefit’[All Fields] AND ‘analysis’[All Fields]) OR ‘cost-benefit analysis’[All Fields] OR (‘cost’[All Fields] AND ‘benefit’[All Fields]) OR ‘cost benefit’[All Fields]) OR (economic evaluation[All Fields] OR economic evaluation,[All Fields] OR economic evaluations[All Fields] OR economic evaluations,[All Fields]) OR cost-utility[All Fields] OR (marginal analyses[All Fields] OR marginal analysis[All Fields]) OR ((‘economics’[Subheading] OR ‘economics’[All Fields] OR ‘cost’[All Fields] OR ‘costs and cost analysis’[MeSH Terms] OR (‘costs’[All Fields] AND ‘cost’[All Fields] AND ‘analysis’[All Fields]) OR ‘costs and cost analysis’[All Fields]) AND benefit$[All Fields]) OR ((‘costs and cost analysis’[MeSH Terms] OR (‘costs’[All Fields] AND ‘cost’[All Fields] AND ‘analysis’[All Fields]) OR ‘costs and cost analysis’[All Fields] OR ‘costs’[All Fields]) AND benefit$[All Fields]) OR (‘cost-benefit analysis’[MeSH Terms] OR (‘cost-benefit’[All Fields] AND ‘analysis’[All Fields]) OR ‘cost-benefit analysis’[All Fields] OR (‘cost’[All Fields] AND ‘effectiveness’[All Fields]) OR ‘cost effectiveness’[All Fields]) OR (‘costs and cost analysis’[MeSH Terms] OR (‘costs’[All Fields] AND ‘cost’[All Fields] AND ‘analysis’[All Fields]) OR ‘costs and cost analysis’[All Fields] OR (‘cost’[All Fields] AND ‘comparison’[All Fields]) OR ‘cost comparison’[All Fields]) OR (cost analyses[All Fields] OR cost analysis[All Fields] OR cost analysis,[All Fields]) OR (costs analyses[All Fields] OR costs analysis[All Fields]) OR (action analyses[All Fields] OR action analysis[All Fields]) OR (action analyses[All Fields] OR action analysis[All Fields]) OR ((‘costs and cost analysis’[MeSH Terms] OR (‘costs’[All Fields] AND ‘cost’[All Fields] AND ‘analysis’[All Fields]) OR ‘costs and cost analysis’[All Fields] OR ‘costs’[All Fields]) AND value[All Fields]) OR ((‘economics’[Subheading] OR ‘economics’[All Fields] OR ‘cost’[All Fields] OR ‘costs and cost analysis’[MeSH Terms] OR (‘costs’[All Fields] AND ‘cost’[All Fields] AND ‘analysis’[All Fields]) OR ‘costs and cost analysis’[All Fields]) AND value[All Fields]) OR cost-feasibility[All Fields] OR cost-acceptability[All Fields] OR (willingness[All Fields] AND pay[All Fields]) OR breakeven[All Fields])) AND ((((web-based[All Fields] AND (‘teaching’[MeSH Terms] OR ‘teaching’[All Fields] OR ‘instruction’[All Fields])) OR (online[All Fields] AND (‘learning’[MeSH Terms] OR ‘learning’[All Fields]))) OR (mobile[All Fields] AND (‘learning’[MeSH Terms] OR ‘learning’[All Fields]))) OR ‘blended learning’[All Fields])

1. **Scopus**

( ( TITLE-ABS-KEY ( cost-benefit ) ) OR ( TITLE-ABS-KEY ( Cost-utility ) ) OR ( TITLE-ABS-KEY ( marginal analys*) ) OR ( TITLE-ABS-KEY ( cost and benefit$) ) OR ( TITLE-ABS-KEY ( costs and benefit$ ) ) OR ( TITLE-ABS-KEY ( cost-comparison$ ) ) OR ( TITLE-ABS-KEY ( cost-analys*) ) OR ( TITLE-ABS-KEY ( costs-analys* ) ) OR ( TITLE-ABS-KEY ( cost-minimi$ation analys*) ) OR ( TITLE-ABS-KEY ( Costs and value ) ) OR ( TITLE-ABS-KEY ( Cost and value ) ) OR ( TITLE-ABS-KEY ( Cost-feasibility ) ) OR ( TITLE-ABS-KEY ( Cost-acceptability ) ) OR ( TITLE-ABS-KEY ( Willingness to pay ) ) OR ( TITLE-ABS-KEY ( Breakeven ) ) OR ( TITLE-ABS-KEY ( economic evaluation ) ) OR ( TITLE-ABS-KEY ( cost-effectiveness ) ) ) AND ( ( TITLE-ABS-KEY ( ‘blended learning’ ) ) OR ( TITLE-ABS-KEY ( elearning ) ) OR ( TITLE-ABS-KEY ( ‘mobile learning’ ) ) OR ( TITLE-ABS-KEY ( ‘online learning’ ) ) ) AND ( ( TITLE-ABS-KEY ( Health Profession$) ) OR ( TITLE-ABS-KEY ( Physical Therap*) ) OR ( TITLE-ABS-KEY ( Physiotherapy) ) OR ( TITLE-ABS-KEY ( General Practitioner$) ) OR ( TITLE-ABS-KEY ( Family practitioner$ ) ) OR ( TITLE-ABS-KEY ( General Physician$) ) OR ( TITLE-ABS-KEY ( General Physician$) ) OR ( TITLE-ABS-KEY ( Hospitalist ) ) OR ( TITLE-ABS-KEY ( Surgeon$) ) OR ( TITLE-ABS-KEY ( Occupational health) ) OR ( TITLE-ABS-KEY (Occupational therap* ) ) OR ( TITLE-ABS-KEY ( Physician$ ) ) OR ( TITLE-ABS-KEY ( Chiropractic) ) OR ( TITLE-ABS-KEY ( Dentist$ ) ) OR ( TITLE-ABS-KEY ( Optometr* ) ) OR ( TITLE-ABS-KEY ( Orthopt* ) ) OR ( TITLE-ABS-KEY ( Pharma* ) ) OR ( TITLE-ABS-KEY ( Podiat*) ) OR ( TITLE-ABS-KEY ( Psycholog* ) ) OR ( TITLE-ABS-KEY ( Serolog*) ) OR ( TITLE-ABS-KEY ( dietitian ) ) OR ( TITLE-ABS-KEY ( Nutrition* ) ) OR ( TITLE-ABS-KEY ( Paramedic* ) ) OR ( TITLE-ABS-KEY ( Community health work$ ) ))

1. **ERIC**

elearning or ‘blended learning’ or ‘online learning’ AND Health Profession$ OR Physical Therap* OR Physiotherapy OR General Practitioner$ OR Family practitioner$ OR General Physician$ OR Family Physician$ OR Hospitalist OR Surgeon$ OR Occupational health OR Occupational therap* OR Physician$ OR Chiropractic OR Dentist$ OR Optometr* OR Orthopt* OR Pharma* OR Podiat* OR Psycholog* OR Serolog* OR dietitian OR Nutrition* OR Paramedic* OR Community health work$ and cost

1. **Web of Science**

TOPIC: (Health Profession$) OR TOPIC: (Physical Therap*) OR TOPIC: (Physiotherapy) OR TOPIC: (General Practitioner$) OR TOPIC: (Family practitioner$) OR TOPIC: (General Physician$) OR TOPIC: (Family Physician$) OR TOPIC: (Hospitalist) OR TOPIC: (Surgeon$) OR TOPIC: (Occupational health) OR TOPIC: (Occupational therap*) OR TOPIC: (Physician$) OR TOPIC: (Chiropractic) OR TOPIC: (Dentist$) OR TOPIC: (Optometr*) OR TOPIC: (Orthopt*) OR TOPIC: (Pharma*) OR TOPIC: (Podiat*) OR TOPIC: (Psycholog*) OR TOPIC: (Serolog*) OR TOPIC: (dietitian) OR TOPIC: (Nutrition*) OR TOPIC: (Paramedic*) OR TOPIC: (Community health work$) Search language=English AND TOPIC: (cost-benefit) OR TOPIC: (Economic evaluation*) OR TOPIC: (Cost-utility) OR TOPIC: (marginal analys*) OR TOPIC: (cost and benefit$) OR TOPIC: (costs and benefit$) OR TOPIC: (Cost-effectiveness) OR TOPIC: (cost-comparison$) OR TOPIC: (cost-analys*) OR TOPIC: (costs-analys*) OR TOPIC: (cost-minimi$ation analys*) OR TOPIC: (cost-minimi$ation analys*) OR TOPIC: (Costs and value) OR TOPIC: (Cost and value) OR TOPIC: (Cost-feasibility) OR TOPIC: (Cost-acceptability) OR TOPIC: (Willingness to pay) OR Search language=English AND TOPIC: (blended learning) OR TOPIC: (online learning) OR TOPIC: (elearning) Search language=English

1. **EMBASE, OVID, GLOBAL HEALTH, HMIC**

(Health Profession$ or Physical Therap* or Physiotherapy or General Practitioner$ or Family practitioner$ or General Physician$ or Family Physician$ or Hospitalist or Surgeon$ or Occupational health or Occupational therap* or Physician$ or Chiropractic or Dentist$ or Optometr* or Orthopt*OR Pharma* or Podiat* or Psycholog* or Serolog* or dietitian or Nutrition* or Paramedic* or Community health work$).mp. [mp=tx, bt, ti, ab, ct, sh, hw, tn, ot, dm, mf, dv, kw, id, cc, nm, kf, px, rx, an, ui] and (((((((((cost-benefit or Economic evaluation* or Cost-utility or marginal analys* or cost) and benefit$) or costs) and benefit$) or Cost-effectiveness or cost-comparison$ or cost-analys* or costs-analys* or cost-minimi$ation analys* or cost-minimi$ation analys* or Costs) and value) or Cost) and value) or Cost-feasibility or Cost-acceptability or Willingness to pay or Breakeven).mp. [mp=tx, bt, ti, ab, ct, sh, hw, tn, ot, dm, mf, dv, kw, id, cc, nm, kf, px, rx, an, ui] AND (‘blended learning’ or ‘online learning’ or elearning).mp. [mp=tx, bt, ti, ab, ct, sh, hw, tn, ot, dm, mf, dv, kw, id, cc, nm, kf, px, rx, an, ui]

1. **PROSPERO**

Limited advanced search capability on database. Used following terms: eLearning OR blended learning OR online learning AND cost* OR economic*

1. **Results Screening**

Abstract Contains trial OR Abstract Contains systematic* AND Abstract Contains online OR Abstract Contains blended OR Abstract Contains web AND Keywords contains education AND Any Field Contains cost*
