# Supplementary material for: Exploring the Cost of eLearning in Health Professions Education: Scoping Review
Source: JMIR Med Educ. 2021 Mar 11;7(1):e13681. doi: 10.2196/13681 (PMC8081275; doi:10.2196/13681)
Supplement: Multimedia Appendix 2 [file mededu_v7i1e13681_app2.docx]

# Multimedia Appendix 2: Eligibility Stage Search Exclusions

| Prefix | No | First Author | Year | Reason for Exclusion | Additional Comments |
| --- | --- | --- | --- | --- | --- |
| EXC | 1 | A Bussieres | 2014 | Not eLearning |  |
| EXC | 2 | A Gardner | 2018 | Not eLearning |  |
| EXC | 3 | A Greech | 2018 | No cost data |  |
| EXC | 4 | A Huhn | 2018 | Not eLearning |  |
| EXC | 5 | A Malfliet | 2018 | No cost data | Suggests cost-effectiveness should be analyzed as further research. |
| EXC | 6 | A Pourmand | 2018 | Not eLearning |  |
| EXC | 7 | A Srivastava | 2014 | Not eLearning |  |
| EXC | 8 | A Woolley | 2013 | Not eLearning |  |
| EXC | 9 | Aggarwal | 2009 | No cost data |  |
| EXC | 10 | Ashurst | 2012 | No cost data | Sites that online methods may be more cost-effective, but requires further study. |
| EXC | 11 | B Fuehrlein | 2016 | Not eLearning |  |
| EXC | 12 | B Naresh | 2015 | No cost data |  |
| EXC | 13 | B Naresh | 2015 | No cost data | Indicates cost a driver but with no specific data points. |
| EXC | 14 | B Yorkgitis | 2017 | Not eLearning |  |
| EXC | 15 | Banks | 2014 | No cost data | Concluded cost-effective, but with no associated results. |
| EXC | 16 | Bateman | 2012 | No cost data |  |
| EXC | 17 | Bellido | 2011 | No cost data |  |
| EXC | 18 | Bitton | 2014 | No cost data | Cost stated as a driver, but with no associated results. |
| EXC | 19 | Boling | 2013 | No cost data |  |
| EXC | 20 | Bowie | 2013 | No cost data | Concluded examination of cost-benefit requires further study. |
| EXC | 21 | Buxton | 2013 | No cost data |  |
| EXC | 22 | C Chan | 2017 | Not eLearning |  |
| EXC | 23 | C Cunningham | 2017 | Not eLearning |  |
| EXC | 24 | C Gay | 2016 | Not eLearning |  |
| EXC | 25 | C Ho | 2018 | No cost data | Concluded could lead to lower cost implementation, however included no data. |
| EXC | 26 | C Lehna | 2014 | No cost data |  |
| EXC | 27 | C Tian | 2014 | No cost data |  |
| EXC | 28 | C Tochel | 2009 | Not eLearning |  |
| EXC | 29 | Chaiyachati | 2014 | Not eLearning |  |
| EXC | 30 | Charman | 2011 | No cost data |  |
| EXC | 31 | Chhabra | 2013 | No cost data | Concluded eLearning more cost-effective via reference and not experimental data. |
| EXC | 32 | Claxton | 2011 | No cost data | States cost-effective means are desirable. |
| EXC | 33 | Colman-Brochu | 2009 | No cost data | Cost stated as a driver, but with no associated results. |
| EXC | 34 | Cothran | 2009 | No cost data | Concluded could lead to lower cost implementation. |
| EXC | 35 | D Andrew | 2008 | No cost data | Concluded cost was a barrier to eLearning uptake. |
| EXC | 36 | D Ettlin | 2016 | No cost data | Concluded could lead to lower cost implementation. |
| EXC | 37 | D McLeod | 2014 | No cost data |  |
| EXC | 38 | D Munafo | 2016 | Not eLearning |  |
| EXC | 39 | D Peterson | 2008 | No cost data |  |
| EXC | 40 | D Smith | 2011 | No cost data |  |
| EXC | 41 | D White | 2008 | Not eLearning |  |
| EXC | 42 | Danielson | 2014 | Not eLearning |  |
| EXC | 43 | Davis | 2014 | No cost data | Cost stated as a driver, but with no associated results. |
| EXC | 44 | Dinleyici | 2013 | Not eLearning |  |
| EXC | 45 | Donald | 2013 | No cost data |  |
| EXC | 46 | Dragovic | 2014 | No cost data |  |
| EXC | 47 | E Dinleyici | 2013 | Not eLearning |  |
| EXC | 48 | E Meinert | 2018 | No cost data |  |
| EXC | 49 | E Murray | 2015 | Not eLearning |  |
| EXC | 50 | E Schneider | 2017 | Not eLearning |  |
| EXC | 51 | E Williamson | 2018 | Not eLearning |  |
| EXC | 52 | E Willignendael | 2005 | Not eLearning |  |
| EXC | 53 | Eng | 2013 | No cost data |  |
| EXC | 54 | Eryilmaz | 2013 | No cost data |  |
| EXC | 55 | F Bishop | 2014 | Not eLearning |  |
| EXC | 56 | F Lobban | 2017 | No cost data |  |
| EXC | 57 | F Pickard | 2014 | No cost data |  |
| EXC | 58 | F Pradel | 2008 | Not eLearning |  |
| EXC | 59 | Fontelo | 2012 | No cost data | Concluded could lead to lower cost implementation. |
| EXC | 60 | G Currie | 2014 | Not eLearning |  |
| EXC | 61 | G Perryer | 2000 | No cost data |  |
| EXC | 62 | Graafland | 2013 | No cost data |  |
| EXC | 63 | Grieff | 2014 | No cost data |  |
| EXC | 64 | Grieff | 2013 | No cost data |  |
| EXC | 65 | Guise | 2012 | No cost data |  |
| EXC | 66 | Hibbert | 2013 | No cost data | Concluded could lead to lower cost implementation. |
| EXC | 67 | Hu | 2009 | No cost data | Concluded cost-effective, but with no associated results. |
| EXC | 68 | Ilic | 2015 | No cost data | Concluded further research on the cost-effectiveness of EBM teaching modalities is required. |
| EXC | 69 | J Cote | 2012 | Not eLearning |  |
| EXC | 70 | J Curtis | 2007 | Not eLearning |  |
| EXC | 71 | J Eriksen | 2018 | Not eLearning |  |
| EXC | 72 | J Fortney | 2012 | Not eLearning |  |
| EXC | 73 | J Kibble | 2011 | No cost data |  |
| EXC | 74 | J Mersereau | 2013 | Not eLearning |  |
| EXC | 75 | J Pechacek | 2015 | Not eLearning |  |
| EXC | 76 | J Place | 2019 | Limited cost data |  |
| EXC | 77 | J Ruiz | 2006 | No cost data | Suggest could be more cost effective, but with no corresponding data |
| EXC | 78 | J Starren | 2002 | No cost data |  |
| EXC | 79 | J Whiteman | 2013 | No cost data |  |
| EXC | 80 | J Yang | 2017 | Not eLearning |  |
| EXC | 81 | K Belogianni | 2018 | No cost data | Intended purpose of study was to introduce cost-effective learning, yet no cost analysis in manuscript |
| EXC | 82 | K Calzone | 2018 | Not eLearning |  |
| EXC | 83 | K Harrington | 2012 | Not eLearning |  |
| EXC | 84 | K Hauer | 2004 | Not eLearning |  |
| EXC | 85 | K Klein | 2012 | No cost data |  |
| EXC | 86 | K long | 2007 | No cost data |  |
| EXC | 87 | K Ly | 2012 | No cost data |  |
| EXC | 88 | K Pfeiffer | 2018 | Not eLearning |  |
| EXC | 89 | K Shen | 2012 | Not eLearning |  |
| EXC | 90 | K Stuber | 2005 | Not eLearning |  |
| EXC | 91 | Kaufmann | 2013 | No cost data |  |
| EXC | 92 | Klein | 2012 | No cost data | Cost stated as a driver, but with no associated results. |
| EXC | 93 | Klien | 2012 | No cost data | Cost stated as a driver, but with no associated results. |
| EXC | 94 | L Leishman | 2013 | No cost data |  |
| EXC | 95 | L Marsh | 2015 | No cost data | Suggest could be more cost effective, but with no corresponding data |
| EXC | 96 | L Moore | 2017 | No cost data |  |
| EXC | 97 | L Yardley | 2010 | No cost data |  |
| EXC | 98 | Lehna | 2014 | No cost data |  |
| EXC | 99 | M Blumenschine | 2018 | Not eLearning |  |
| EXC | 100 | M Brunette | 2015 | Not eLearning |  |
| EXC | 101 | M Hertz | 2008 | No cost data | Concluded could lead to lower cost implementation, however included no data. |
| EXC | 102 | M Li | 2016 | No cost data |  |
| EXC | 103 | M Morgan | 2014 | No cost data |  |
| EXC | 104 | M Price | 2009 | Not eLearning |  |
| EXC | 105 | M Rasura | 2014 | Limited cost data |  |
| EXC | 106 | M Tchou | 2017 | Not eLearning |  |
| EXC | 107 | M Willis | 2016 | No cost data |  |
| EXC | 108 | Manners | 2013 | No cost data |  |
| EXC | 109 | Martin | 2014 | No cost data |  |
| EXC | 110 | McLeod | 2012 | No cost data |  |
| EXC | 111 | McVey | 2013 | No cost data |  |
| EXC | 112 | Mittelman | 2014 | No cost data | Concluded could lead to low-cost implementation. |
| EXC | 113 | Mobley | 2011 | No cost data |  |
| EXC | 114 | Myers | 2009 | No cost data | Cost stated as a driver, but with no associated results. |
| EXC | 115 | N Henrikson | 2014 | Not eLearning |  |
| EXC | 116 | N Kohle | 2015 | No cost data |  |
| EXC | 117 | N Milic | 2016 | No cost data |  |
| EXC | 118 | N Rocha-Pereira | 2015 | No cost data |  |
| EXC | 119 | O Simmons | 2018 | Not eLearning |  |
| EXC | 120 | Okrainec | 2010 | No cost data | Concluded cost-effective, but with no associated results. |
| EXC | 121 | P Bowie | 2013 | Not eLearning | Concluded examination of cost-benefit requires further study. |
| EXC | 122 | P Butow | 2018 | Not eLearning |  |
| EXC | 123 | P Garcia | 2009 | No cost data |  |
| EXC | 124 | P McDonald | 2017 | No cost data |  |
| EXC | 125 | P Nambisan | 2010 | No cost data |  |
| EXC | 126 | P Reynolds | 2008 | No cost data |  |
| EXC | 127 | Parker | 2010 | No cost data | Cost stated as a driver, but with no associated results. |
| EXC | 128 | Parker | 2010 | No cost data | Cost stated as a driver, but with no associated results. |
| EXC | 129 | Patterson | 2011 | No cost data |  |
| EXC | 130 | Phillippi | 2010 | No cost data |  |
| EXC | 131 | Pinto | 2008 | No cost data | Concluded could lead to lower cost implementation. |
| EXC | 132 | Piorkowski | 2013 | No cost data |  |
| EXC | 133 | Platz | 2010 | No cost data | Concluded could lead to lower cost implementation. |
| EXC | 134 | Pletcher | 2011 | No cost data |  |
| EXC | 135 | R Carrick | 2017 | No cost data | Cost stated as a driver, but with no associated results. |
| EXC | 136 | R Enserick | 2014 | Not eLearning |  |
| EXC | 137 | R Hughes | 2018 | Not eLearning |  |
| EXC | 138 | R Pettit | 2017 | Not eLearning |  |
| EXC | 139 | R Tamler | 2012 | No cost data |  |
| EXC | 140 | Rogers | 2011 | No cost data |  |
| EXC | 141 | S Claudel | 2018 | Not eLearning |  |
| EXC | 142 | S Cooper | 2016 | Not eLearning |  |
| EXC | 143 | S Glegg | 2016 | No cost data |  |
| EXC | 144 | S Jennings | 2014 | Not eLearning |  |
| EXC | 145 | S Nobis | 2018 | Not eLearning |  |
| EXC | 146 | S Shah | 2012 | Not eLearning |  |
| EXC | 147 | S Sheridan | 2013 | Not eLearning |  |
| EXC | 148 | Saker | 2010 | No cost data |  |
| EXC | 149 | Scott | 2013 | No cost data | Cost stated as driver, but with no associated results. |
| EXC | 150 | Shaikh | 2012 | Limited cost data | Incomplete costing details. |
| EXC | 151 | Stevenson | 2011 | No cost data |  |
| EXC | 152 | Stewart | 2010 | No cost data |  |
| EXC | 153 | Sung | 2008 | No cost data | Concluded could lead to lower cost implementation. |
| EXC | 154 | T Coughlan | 2015 | No cost data | Cost stated as a driver, but with no associated results. |
| EXC | 155 | T Deliens | 2016 | Not eLearning |  |
| EXC | 156 | T Hartranft | 2017 | Not eLearning |  |
| EXC | 157 | T Krebs | 1999 | Limited cost data | While provided cost per learner, no details as to how calculated. Indicated online costs lower than face to face. |
| EXC | 158 | T Luckett | 2018 | No cost data | Protocol with education secondary area of analysis without explicit detail as to how costs would be analyzed. |
| EXC | 159 | T Pascual | 2013 | No cost data |  |
| EXC | 160 | Trocky | 2011 | No cost data | Cost stated as a driver, but with no associated results. |
| EXC | 161 | Walsh | 2014 | No cost data |  |
| EXC | 162 | Wisner | 2008 | No cost data | Concluded cost-effective, but with no associated results. |
| EXC | 163 | Y AlJamal | 2018 | Not eLearning |  |
| EXC | 164 | Y Erard | 2018 | Not eLearning |  |
| EXC | 165 | Y Ramallo-Farina | 2015 | Not eLearning |  |
| EXC | 166 | Y Sung | 2008 | No cost data |  |
| EXC | 167 | Z Ma | 2008 | No cost data |  |
| EXC | 168 | Z Pruitt | 2017 | Not eLearning |  |
